# Preschool children aged 4 to 5 years show discomfort with trypophobic images

**DOI:** 10.1038/s41598-023-29808-1

**Published:** 2023-02-16

**Authors:** Chiharu Suzuki, Nobu Shirai, Kyoshiro Sasaki, Yuki Yamada, Tomoko Imura

**Affiliations:** 1 Department of Psychology, Faculty of Integrated Arts and Social Sciences, Japan Women’s University, Tokyo, Japan; 2 Department of Psychology, Faculty of Humanities, Niigata University, Niigata, Japan; 3 Faculty of Informatics, Kansai University, Osaka, Japan; 4 Faculty of Arts and Science, Kyushu University, Fukuoka, Japan; 5 Department of Psychology, Faculty of Integrated Arts and Social Sciences, Japan Women’s University, 2-8-1, Mejirodai, Bunkyo-Ku, Tokyo, 112-8681 Japan; 6 Present Address: Department of Psychology, College of Contemporary Psychology, Rikkyo University, Saitama, Japan

**Keywords:** Human behaviour, Ageing

## Abstract

The fear or disgust of clustered patterns, such as honeycomb or lotus seed pods, is known as trypophobia. A previous developmental study reported that 4-year-old children prefer neutral images over clustered images. However, whether those results indicated higher rating scores for trypophobic images has been controversial. In this study, we examined discomfort with trypophobic images in adults and children aged 4–9 years using an identical experimental procedure. A modified rating scale applicable for children was used that was based on the established Trypophobia Scale for adults. The participants were required to rate five trypophobic and five neutral images on four rating items (disgusting, fear, feel itchiness, and like) on a 4-point scale ranging from 1 (not at all) to 4 (very much). The participants in all age groups indicated higher rate scores for trypophobic images than for neutral images in terms of ‘disgust’, ‘fear’, and ‘feeling itchiness’, whereas they indicated higher scores for neutral images than for trypophobic images in terms of ‘like’. These results suggest that children aged 4–5 years have responses comparable to the responses of adults with respect to trypophobic and neutral images; thus, trypophobia appears to emerge at least by the age of 4–5 years.

## Introduction

Discomfort toward a cluster of holes or circles, such as lotus seed pods or a honeycomb, is known as “trypophobia”. The word trypophobia comprises the ancient Greek word “trypa (τρύπα)”, meaning ‘to make a hole’, and the English word “phobia” (from the ancient Greek ‘φόβος’), meaning “fear”^1^. Cole and Wilkins^2^ were the first to investigate trypophobia and reported that 11% of men (n = 19) and 18% of women (n = 195) showed an aversive response, although it has yet to be recognized as a phobia in the Diagnostic and Statistical Manual of Mental Disorders, Fifth Edition (DSM-5)^3^. Empirical research has revealed factors that induce aversion to trypophobic images in nonclinical adults.

Previous research has suggested that the spatial frequency characteristics of trypophobic images promote visual discomfort^2,4^. Cole and Wilkins^2^ performed spectral analysis of a set of spatial frequency components in trypophobic and neutral images (containing a hole but not aggregates) to investigate the relationship between discomfort and the spectral composition; they found that the decrease in component amplitude was approximately proportional to the reciprocal of spatial frequency (1/*f*) in the neutral images, whereas the contrast energy of the mid-range spatial frequencies (1.5–18 cycles per degree) was higher in the trypophobic images than in the neutral images. They also found that images of venomous animals (e.g., snakes and spiders) have spatial frequency characteristics similar to the characteristics of trypophobic images. Le, Cole, and Wilkins^5^ examined the effects of the number of objects (cluster size: 16, 64, or 256) in an aggregate image on discomfort; they showed that images with larger cluster sizes, which potentially contain more high spatial frequency components (such that the amplitude spectrum of image deviates from 1/*f*), induce more discomfort.

Although the spectral composition of spatial frequency components in images can contribute to the induction of trypophobic responses, other factors have effects on trypophobia. For instance, Le et al.^5^ examined the effects of the shape of circular objects (either concave or convex shapes defined by a depth cue of shading, or a mixture of the two shapes); they showed that discomfort was higher for clusters of mixed shapes than for clusters of concave shapes that resembled holes. Their results suggest that the cluster of holes do not induce more discomfort, compared with a cluster of convex shapes or a mixed cluster of convex and concave shapes. Because the difference in convex/concave shapes appeared not to have affected the spectral characteristics of the image in^5^, the difference in reported discomfort among the three images (convex, concave, and a mixture of the two) could not be explained by the spectral characteristics of the images. In addition, Pipitone and DiMattina^6^ independently manipulated the amplitude and phase spectrum of the spatial frequency components in trypophobic images and directly compared such aspects to investigate the effects of the spectrum information on the ratings of discomfort; they reported that the phase information has a greater role in the induction of trypophobia than does the amplitude information. Therefore, the energy spectrum of a set of spatial frequency components in trypophobic images may not be the only factor responsible for inducing visual discomfort.

Furthermore, recent studies have shown that trypophobic traits are related to both visual discomfort and core disgust, which is an aversion to infectious diseases^7,8^. Some researchers have argued that trypophobia may originate from an overgeneralized avoidance of infectious disease [e.g.,^8,9^]. Yamada and Sasaki^9^ investigated the relationship between trypophobia and skin diseases, focusing on infections with multiple circular rashes. Despite the preliminary nature of the investigation, they found that people with a history of skin diseases exhibited more discomfort with trypophobic images than did people without such a history. These studies suggest that trypophobia may remind people of infectious diseases, such as skin diseases.

Despite the divergent findings thus far regarding the presence of trypophobia in adult participants, few studies have been published concerning trypophobic discomfort in children; thus, it remains unclear as to how trypophobic discomfort arises during development. Can, Zhuoran, and Zheng^10^ conducted pioneering developmental research regarding trypophobic discomfort, in which they examined preferences for trypophobic and neutral images in 4-year-olds using a self-report task and the Preschool-Single Category Implicit Association Test; they showed three types of image-pairs to children for the self-report task, such as trypophobic vs. neutral images, colored photos of venomous vs. non-venomous animals, and line drawings of venomous vs. non-venomous animals. The children were then asked to choose their preferred image from each pair. The results indicated that the children avoided choosing the trypophobic images and the photos of venomous animals, whereas there was no significant difference in preference for the line drawings of the venomous and non-venomous animals, suggesting that visual features contained in trypophobic images may affect discomfort. In the same study, Can et al.^10^ used the Preschool-Single Category Implicit Association Test to examine whether trypophobic images are more likely to be associated with venomous animals than with non-venomous animals. The three categories (trypophobic images, venomous animals, and non-venomous animals) were assigned to two response buttons (e.g., one key for the response to trypophobic images and venomous animals, and the other key for the response to non-venomous animals). The children were asked to press the button corresponding to the image. If there were associations between trypophobic images and venomous animals, the response times should have been faster when the responses to those images were assigned the same button. The results indicated no significant association between the trypophobic images and the photos of venomous animals, suggesting that avoidance of the trypophobic images was not a result of learned associations with toxic risks. Although Can et al.^10^ demonstrated that 4-year-old children prefer a neutral image to a trypophobic image, they did not directly assess discomfort toward trypophobic images in children. Moreover, the small number of images (four images for each of the trypophobic and neutral image categories) used by Can et al.^10^ may have weakened their results.

The aim of the present study was to more directly investigate trypophobic tendencies in children with a large number of images. We asked adults and children aged 4–9 years to estimate their discomfort with various images (20 trypophobic images and 20 neutral images) on a 4-point Likert scale. We hypothesized that if trypophobic tendencies occur during both childhood and adulthood, then the rating scores of the items regarding unpleasantness (i.e., ‘fear’, ‘disgusting’, and ‘feel itchiness’) and pleasantness (‘like’) should be higher and lower, respectively, for the trypophobic images than for the neutral images.

## Methods

### Participants

The participants were 4–9-year-old Japanese children and Japanese adults. They were recruited via flyers distributed at public health centers in Niigata City, Japan, and through the website of the research laboratory of the Department of Psychology, Niigata University. Families who responded were recruited to the study.

Table 1 shows the age and gender distribution of the participants. Four additional children participated, but were not included in the final sample because their responses were not consistent between trials (*n* = 2) or within trials (*n* = 2) due to poor verbal skills. Notably, of these four children, two of the children were unable to rate items in some images; thus, those responses (four in total) were excluded. Additionally, an adult was excluded due to the inability to continue with the experiment because she found the images too aversive.

**Table 1:** Age and gender distribution of each age group.

Age (in year)				Gender		
Age group	M	SD	Range	Males	Females	Total
4–5-year-old	4.99	0.55	4.05–5.95	9	11	20
6–7-year-old	7.05	0.54	6.03–7.97	6	14	20
8–9-year-old	9.00	0.60	8.03–9.90	8	12	20
Adults	20.95	0.89	19–22	10	10	20

The sample size (*n* = 20 per age group) was determined as follows. To our knowledge, no similar study has been performed; thus, it was difficult to estimate reasonable effect sizes required to calculate the sample size. As an alternative, we adopted “heuristics” criteria to determine the sample size^11^ and followed the guidance of Simmons, Nelson, and Simonsohn^12^, who recommended a sample size of ≥ 20 for each experimental condition to reduce false-positive results. Ultimately, we used a sample size of 20 individuals for each age group.

## Ethics statement

Written informed consent for study participation was obtained before the experiment. The child participants were requested to provide written informed assent if they could sign their own name; each child’s parents provided written informed consent for the child to participate. Moreover, written informed consent was collected from adult participants. Before the participants (and the parents of child participants) provided informed consent (or assent, as described above), they were shown thumbnail images of the stimuli images. Then, they were advised that if they felt strong discomfort from the images, they could withdraw their participation at any time before/after starting the experiment. All experiments were conducted in accordance with the Declaration of Helsinki. This study was approved by the Ethics Committee for Human Research of Niigata University, Japan (approval no. 2017-0359 v3).

## Stimulus and apparatus

The visual stimuli were presented, and the rating scale assessments and recording of responses were performed using Psychopy3 v3.0.5.^13^ on a laptop computer (MacBook Pro, Apple, 15.4 inches, 331.3 × 207.2 mm). The computer was positioned on the center of the desk and a chair was placed in front of the computer and the desk. The participant sat on the chair with a viewing distance of approximately 60 cm. No supportive equipment (e.g., a chin/head rest) was used.

In total, 40 images from Le et al.^5^ were used as the visual stimuli: 20 trypophobic images (e.g., lotus seed pods and honeycombs), which are clusters of circle or holes that induce trypophobic discomfort, and 20 neutral images (e.g., front view of a cannon and golf cup), which did not induce trypophobic discomfort, but had the common feature of ‘holes’. In each trial, one of the images (512 × 512 pixels in width and height, respectively) appeared at the center of the computer screen with a grey background. An additional neutral image (e.g., sunflower) was used for practice trials. A 4-point Likert scale (800 × 100 pixels in width and height, respectively) was presented immediately below the image.

## Procedure

The experiment was conducted in a darkened room. An experimenter and a participant (and the participant’s parent, if the participant was a child) were in the room. Generally, the child and parent were separated by an opaque partition in the experimental room. However, if the child wished to remain with the parent during the experiment, the parent was allowed to stand behind the child over the partition. In this scenario, the parent was asked to refrain from talking to the child.

Each participant was shown 10 images: 5 trypophobic images and 5 neutral images. The 5 trypophobic and 5 neutral images were randomly selected from the 20 trypophobic and the 20 neutral images, respectively, before the experiment. Participants were required to rate each of the 10 images using one of four rating items: ‘disgusting’, ‘fear’, ‘feel itchiness’, and ‘like’. These four rating items were adopted from the Japanese version of the Trypophobia Questionnaire, which measures the sensitivity of trypophobia^14^. The three items (‘disgusting’, ‘fear’, and ‘feel itchiness’) were chosen because these items are easily understood by young children. The other item (‘like’) was chosen as a reverse item.

A 4-point Likert scale with four labels, ‘not at all,’ ‘not much,’ ‘a little’, and ‘very much’, was used for the rating task. These four labels were chosen in accordance with works by Oda^15^ and Miyashita^16^. Oda^15^ investigated the understanding of quantitative expressions in Japanese used in rating scales from 9-year-olds to adults; the results showed that younger children have more difficulty classifying ‘neither’ and ‘not much’. Thus, as in the study by Miyashita^16^, we omitted the label corresponding to ‘neither’ from the rating scale.

The rating task was conducted using a blocked design, in which each experimental block consisted of four rating trials (fear, disgust, feel itchiness, and like) for each of the 10 images. The order of the rating trials in each block was randomized; the order of the 10 blocks was also randomized. When the participants pressed the return key of the computer to begin an experimental block, an image and the 4-point rating scale appeared on the computer screen. The participants rated their impression of the image by manipulating the trackpad of the computer and clicking the rating scale. When the participant pressed the return key, the answer was confirmed (the answer could be changed as many times as desired before pressing the key). This was followed by the presentation of the next rating item. After the four ratings, a new experimental block was initiated. Before initiation of the 10 experimental blocks, the participants engaged in a practice block with one image (an image of a sunflower). Therefore, each participant engaged in 11 blocks (1 practice block and 10 experimental blocks).

The experimenter orally supported the child participants who had difficulty reading the text. For example, when assessing the ‘like’ rating, the experimenter asked the participant, ‘Do you like this picture or not?’ Then, if their oral response was ‘Yes’, the experimenter asked the participant ‘How much do you like it?’, and confirmed the oral response (e.g., ‘a little’ or ‘very much’). The experimenter also helped the participants to use the trackpad and read the text. The scores ranged from 1 to 4, from strongly negative (i.e., not at all) to strong affirmation (i.e., very much). We calculated the mean rating score for the trypophobic images (five images) and the neutral images (five images), respectively, for each participant.

## Data analysis

We computed the mean of the scores for each item per condition. The alpha level was 0.05 for all statistical tests. We conducted a two-way mixed analysis of variance on each of the mean scores (disgust, fear, feel itchiness, and like) with image type (trypophobic and neutral) as a within-group factor and age (4–5 years, 6–7 years, 8–9 years, and adult) as a between-groups factor. If the interaction was significant, we performed a simple main effects tests. When the main effect and simple main effects of the age group were significant, we identified significantly different pairs using Ryan’s method of multiple comparisons^17^. We report *F*-values, *t*-values, *p*-values, and effect sizes (i.e., *η*^2^ and Cohen’s *d*) (see Table 2 for details).

**Table 2:** Results of two-way mixed analysis of variance of the mean scores for (a) disgust, (b) fear, (c) feel itchiness, and (d) like, with image type (trypophobic and neutral) as a within-group factor and age group (4–5 years, 6–7 years, 8–9 years, and adult) as a between-groups factor.

(a) Disgusting						
Age group	Image type	M	SD			
4–5 yrs	Trypophobic	2.343	0.936			
4–5 yrs	Neutral	1.690	0.624			
5–6 yrs	Trypophobic	2.020	0.802			
6–7 yrs	Neutral	1.450	0.513			
7–8 yrs	Trypophobic	2.630	0.762			
8–9 yrs	Neutral	1.170	0.212			
Adults	Trypophobic	2.580	0.767			
Adults	Neutral	1.100	0.173			

Source	Sum of squares	df	Mean Square	F	p	η^2^
Table of analysis of variance						
A: Age group	1.6553	3	0.5518	0.8350	0.4789	0.0137
S × A	50.2277	76	0.6609			
B: Image type	43.3160	1	43.3160	181.6320	0.0000****	0.3588
A × B	7.4105	3		10.3580	0.0000****	0.0614
S × A × B	18.1247	76	0.2385			
Total	120.7342353	159				
Simple main effect						
A (Trypophobic)	4.656344	3	1.5521147	3.452	0.0181*	0.077875173
A (Neutral)	4.4094998	3	1.4698333	3.269	0.0230*	0.073746819
Error		152	0.4496867			
B (4–5 yrs)	4.2575625	1	4.2575625	17.853	0.0001****	0.071205739
B (6–7 yrs)	3.2490001	1	3.2490001	13.624	0.0004****	0.054338005
B (8–9 yrs)	21.3159999	1	21.3159999	89.382	0.0000****	0.356500117
B (adults)	21.9040002	1	21.9040002	91.847	0.0000****	0.366334146

Multiple comparison (Ryan's method)						
Pair	t	*p*	r			
Means on age group (Trypophobic)						
8–9 yrs to 6–7 yrs	2.877	0.0045985*	0.23			
8–9 yrs to 4–5 yrs	1.356	0.1771864 n.s	0.11			
Adults to 6–7 yrs	2.641	0.0091353*	0.21			
8–9 yrs to adults	0.236	0.8139174 n.s	0.02			
Adults to 4–5 yrs	1.12	0.2644907 n.s	0.09			
4–5 yrs to 6–7 yrs	1.521	0.1303852 n.s	0.12			

(b) Fear						
Age group	Image type	M	SD			
4–5 yrs	Trypophobic	2.075	0.800			
4–5 yrs	Neutral	1.700	0.560			
5–6 yrs	Trypophobic	1.790	0.784			
6–7 yrs	Neutral	1.340	0.594			
7–8 yrs	Trypophobic	1.860	0.741			
8–9 yrs	Neutral	1.210	0.349			
Adults	Trypophobic	2.150	0.672			
Adults	Neutral	1.170	0.203			

Source	Sum of squares	df	Mean square	F	p	η^2^
Table of analysis of variance						
A: Age group	3.0557	3	1.0185625	1.7910	0.1560	0.0400
S × A	43.2143	76	0.5686086			
B: Image type	15.0676	1	15.0676	62.0260	0.0000****	0.1838
A × B	2.1927	3	0.7309	3.0090	0.0354*	0.0300
S × A × B	18.4622	76	0.2429243			
Total	81.9924369	159				
Simple main effect						
A (Trypophobic)	1.7584	3	0.586125	1.444	0.2321	0.078120055
A (Neutral)	3.49000	3	1.1633333	2.867	0.0385*	0.155051677
Error		152	0.4057664			
B (4–5 yrs)	1.40625	1	1.40625	5.789	0.0186*	0.062476052
B (6–7 yrs)	2.0250001	1	2.0250001	8.336	0.0051**	0.089965559
B (8–9 yrs)	4.2249998	1	4.2249998	17.392	0.0001****	0.187705817
B (adults)	9.6039998	1	9.6039998	39.535	0.0000****	0.42668088
Error		76	0.2429243			

Multiple comparison (Ryan's method)						
Pair	t	*p*	r			
Means on age group (Neutral)						
4–5 yrs to adults	2.631	0.0093877 n.s	0.21			
4–5 yrs to 8–9 yrs	2.433	0.016155 n.s	0.19			
6–7 yrs to adults	0.844	0.40003 n.s	0.17			
4–5 yrs to 6–7 yrs	1.787	0.0759037 n.s	0.14			
6–7 yrs to 8–9 yrs	0.645	0.5196638 n.s	0.05			
8–9 yrs to adults	0.199	0.8428615 n.s	0.02			

(c) Feel itchiness						
Age group	Image type	M	SD			
4–5 yrs	Trypophobic	2.050	0.802			
4–5 yrs	Neutral	1.707	0.611			
5–6 yrs	Trypophobic	1.390	0.628			
6–7 yrs	Neutral	1.270	0.444			
7–8 yrs	Trypophobic	1.400	0.566			
8–9 yrs	Neutral	1.060	0.180			
Adults	Trypophobic	1.590	0.542			
Adults	Neutral	1.060	0.143			

Source	Sum of squares	df	Mean square	F	*p*	*η*^2^
Table of analysis of variance						
A: Age group	10.4769	3	3.4923072	7.7190	0.0001****	0.1700
S × A	34.3862	76	0.4524498			
B: Image type	4.4388907	1	4.4388907	30.3670	0.0000****	0.1838
A × B	0.8431719	3	0.2811	1.9230	0.1330	0.0138
S × A × B	11.1092	76	0.1461735			
Total	61.2543596	159				

Multiple comparison (Ryan's method)						
Pair	t	*p*	r			
Means on age group						
4–5 yrs to 8–9 yrs	4.313	0.0000478*	0.44			
4–5 yrs to adults	3.682	0.0004312*	0.39			
6–7 yrs to 8–9 yrs	0.665	0.5081529 n.s	0.08			
4–5 yrs to 6–7 yrs	3.648	0.0004814*	0.39			
6–7 yrs to adults	0.033	0.9735681 n.s	0.00			
Adults to 8–9 yrs	0.632	0.5295328 n.s	0.06			

(d) Like						
Age group	Image type	M	SD			
4–5 yrs	Trypophobic	2.140	0.961			
4–5 yrs	Neutral	2.730	0.904			
5–6 yrs	Trypophobic	1.800	0.559			
6–7 yrs	Neutral	2.110	0.706			
7–8 yrs	Trypophobic	1.540	0.502			
8–9 yrs	Neutral	2.160	0.508			
Adults	Trypophobic	1.870	0.426			
Adults	Neutral	2.840	0.609			

Source	Sum of squares	df	Mean Square	F	*p*	η^2^
Table of analysis of variance						
A: Age group	10.0507	3	3.3502499		4.5670	0.0054**
S × A	55.7490	76	0.7335395			
B: Image type	15.5003	1	15.5003	71.2870	0.0000****	0.1500
A × B	2.1947	3	0.7316	3.3650	0.0229*	0.02
S × A × B	16.5250	76	0.2174			
Total	100.0197501	159				
Simple main effect						
A (Trypophobic)	3.64950	3	1.2164999	2.558	0.0572+	0.121891735
A (Neutral)	8.59600	3	2.8653333	6.026	0.0007****	0.287102732
Error		152	0.4754868			
B (4–5 yrs)	3.4810003	1	3.4810003	16.009	0.0001****	0.116263927
B (6–7 yrs)	0.961	1	0.961	4.42	0.0388*	0.032096991
B (8–9 yrs)	3.844	1	3.844	17.679	0.0001****	0.128387963
B (adults)	9.4089996	1	9.4089996	43.273	0.0000****	0.314256579
Error		76	0.2174342			

Multiple comparison (Ryan's method)						
Pair	t	*p*	r			
Means on age group						
4–5 yrs to 8–9 yrs	3.055	**0.0031066**	0.33			
4–5 yrs to adults	2.506	0.014335	0.28			
6–7 yrs to 8–9 yrs	2.637	**0.0101405**	0.29			
4–5 yrs to 6–7 yrs	0.418	0.6773241	0.05			
6–7 yrs to adults	2.089	0.0400889	0.23			
Adults to 8–9 yrs	0.548	0.5851152	0.06			
Means on age group (Neutral)						
Adults to 6–7 yrs	3.348	0.001027*	0.26			
Adults to 8–9 yrs	3.118	0.0021751*	0.25			
4–5 yrs to 6–7 yrs	2.843	0.0050794*	0.22			
Adults to 4–5 yrs	0.504	0.6146714 n.s	0.04			
4–5 yrs to 8–9 yrs	2.614	**0.009849**	0.21			
8–9 yrs to 6–7 yrs	0.229	0.8189453 n.s	0.02			

+ *p* < .10, * *p* < .05, ** *p* <.01, *** *p* < .005, **** *p* < .001

MSe = 0.405766, df = 76, significance level = 0.050000

MSe = 0.475487, df = 152, significance level = 0.050000

Significant values are in [bold].

## Results

**Figure 1:**
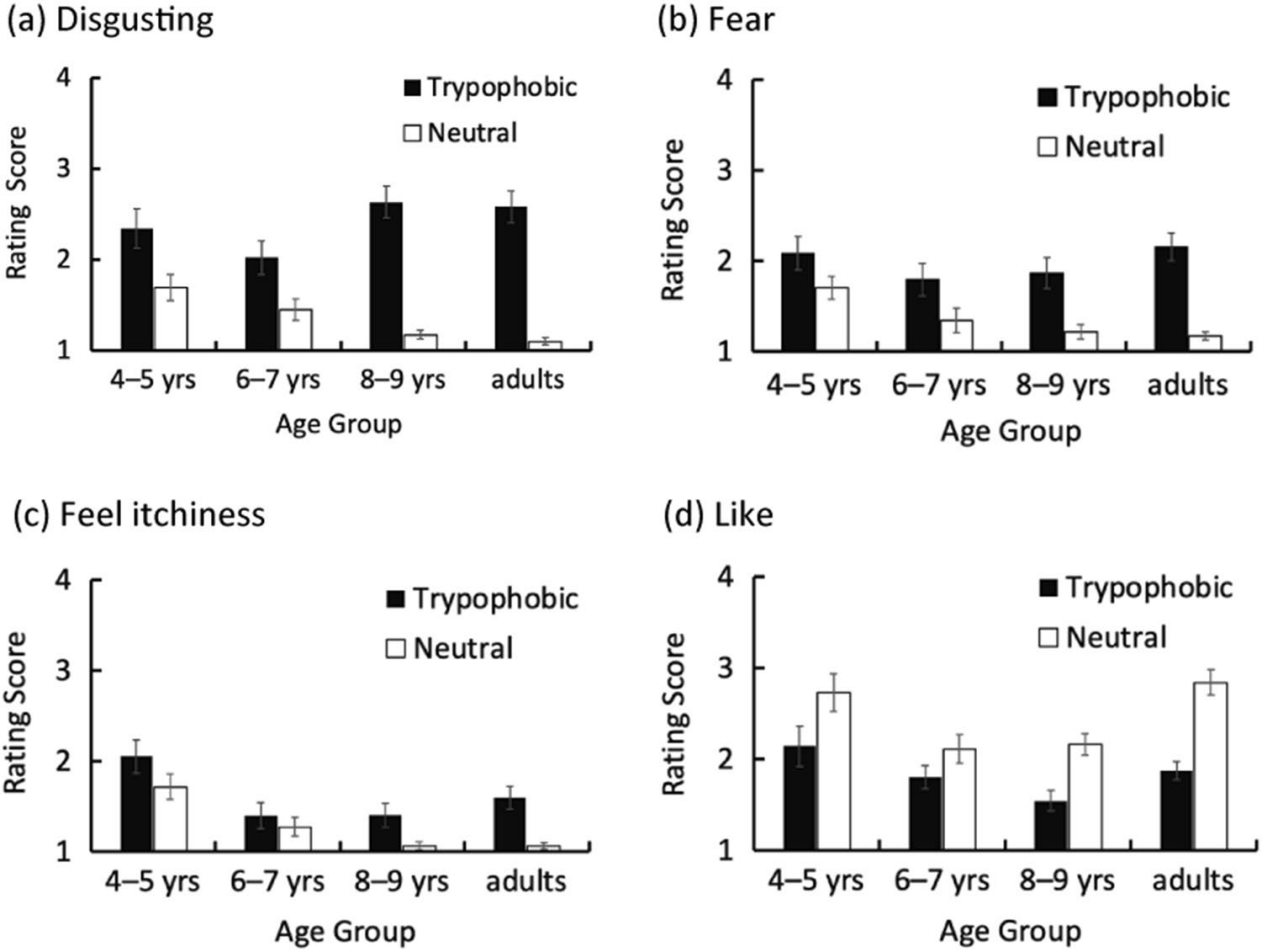
Mean ratings across age groups for the ‘disgusting’ (**a**), ‘fear’ (**b**), ‘feel itchiness’ (**c**), and ‘like’ (**d**) items. The black bars represent the results for the trypophobic images and the white bars represent the results for the neutral image. The error bars represent the mean ± 1 standard error of the mean (SEM).

The mean ratings across age groups are shown in Fig. 1a–d. As shown in Fig. 1a, ‘disgusting’ rating scores were significantly higher for the trypophobic condition compared with the control condition, regardless of age group, i.e., there was a significant main effect of image type (*F*(1, 76) = 181.63, *p* < 0.001, *η*^2^ = 0.36) but not age group (*F* (3, 76) = 0.84, *p* = 0.48, *η*^2^ = 0.01). In addition, the difference in scores between the trypophobic and control conditions increased with age, i.e., there was a significant interaction between image type and age group (*F*(3, 76) = 10.36, *p* < 0.001, *η*^2^ = 0.06), and a significant simple main effect of age group for both image types (trypophobic: *F*(3, 76) = 3.45, *p* = 0.02, *η*^2^= 0.08; neutral: *F*(3, 76) = 3.27, *p* = 0.02, *η*^2^ = 0.07). The multiple comparisons for the simple main effect of age group in the trypophobic condition revealed that the mean ratings of 8–9-year-olds and adults were significantly higher than the mean ratings of 6–7-year-olds (8–9-year-olds: *t*(152) = 2.88, *p* = 0.005, *d* = 0.63; adults: *t*(152) = 2.64, *p* = 0.009, *d* = 0.56). The simple main effect of image type was also significant for all age groups (4–5-year-olds: *F*(1, 76) = 17.85, *p* < 0.001, *η*^2^ = 0.07; 6–7-year-olds: *F*(1, 76) = 13.62, *p* < 0.001, *η*^2^ = 0.05; 8–9-year-olds: *F*(1, 76) = 89.38, *p* < 0.001, *η*^2^ = 0.36; adults: *F*(1, 76) = 91.85, *p* < 0.001, *η*^2^ = 0.37).

As shown in Fig. 1b, the ‘fear’ scores for the trypophobic condition were higher than those for the control condition for all age groups, i.e., there was a main effect of image type (*F*(1, 76) = 62.03, *p* < 0.001, *η*^2^ = 0.18), and a significant interaction effect (*F*(3, 76) = 3.01, *p* = 0.04, *η*^2^ = 0.03), whereas the main effect of age was not significant (*F*(3, 76) = 1.79, *p* = 0.16). The simple main effect of image type was significant in all age groups (4–5-year-olds: *F*(1, 76) = 5.79, *p* = 0.05, *η*^2^ = 0.06; 6–7-year-olds: *F*(1, 76) =8.34, *p* = 0.01, *η*^2^ = .09; 8–9-year-olds: *F*(1, 76) = 17.39, *p* < 0.001, *η*^2^ = 0.19; adults:* F*(1, 76) = 39.54, *p* < 0.001, *η*^2^ = 0.43). On the other hand, the simple main effect of age was significant only for the neutral image (*F*(3, 76) = 2.89, *p* = 0.04, *η*^2^ = 0.16), where ratings therefor decreased with age.

Figure 1c also shows that the ‘feel itchiness’ scores for the trypophobic condition were higher than those for the control condition in all age groups. The main effect of image type (*F*(1, 76) = 30.37, *p* < 0.001, *η*^2^ = 0.07) and the main effect of age group (*F*(3, 76) = 7.72, *p* < 0.001, *η*^2^ = 0.17) were significant, whereas the interaction was not significant (*F*(3, 76) = 1.92, *p* = 0.13, *η*^2^ = 0.01). Multiple comparisons for the main effect of age revealed that the mean ratings of 4–5-year-olds were significantly higher than the mean ratings of all other age groups (6–7-year-olds: *F*(1, 76) = 3.65, *p* < 0.001, *η*^2^ = 0.39; 8–9-year-olds: *F*(1, 76) = 4.31, *p* < 0.001, *η*^2^ = 0.44; adults: *F*(1, 76) = 3.68, *p* < 0.001, *η*^2^ = 0.39).

Unlike the other items, the scores for the ‘like’ item for the trypophobic condition were lower than those for the control condition (Fig. 1d). The main effect of image type (*F*(1, 76) = 71.29, *p* < 0.001, *η*^2^ = 0.15) and the main effect of age (*F*(3, 76) = 4.57 *p* = 0.01, *η*^2^ = 0.10) were significant. The interaction between these two factors was also significant (*F* (3, 76) = 3.37, *p* = 0.02, *η*^2^ = 0.02). The simple main effect of image type was significant for all age groups (4–5-year-olds: *F*(1, 76) = 16.01, *p* < .001, *η*^2^ = 0.12; 6–7-year-olds: *F*(1, 76) = 4.42, *p* = 0.04, *η*^2^ = .03; 8–9-year-olds: *F*(1, 76) = 17.68, *p* < 0.001, *η*^2^ = 0.13; adults: *F*(1, 76) = 43.27, *p* <0 .001, *η*^2^ = 0.31). These results suggest that the ‘like’ scores for trypophobic images were lower than those for control images in all age groups, although the mean scores of 4–5-year-olds and adults were significantly higher than those of 8–9-year-olds.

In summary, the ratings for the trypophobic image were significantly higher than those for the control images, while the main effects of age were significant only for the ‘feel itchiness’ and ‘like’ items. The interactions between age and image type were significant for all rating items without the ‘feel itchiness’ item, and the scores for the trypophobic image were significantly higher than those for the control images in all age groups.

## Discussion

In this study, we examined whether adults and children aged 4–9 years experienced discomfort from trypophobic images. The participants rated emotional impressions (‘disgusting’, ‘fear’, ‘feel itchiness’, and ‘like’ items) of the trypophobic and neutral images on a 4-point scale. The results showed that the ratings were higher for trypophobic images than for neutral images in all age groups with respect to the ‘disgusting’, ‘fear’, and ‘feel itchiness’ items; the ratings were higher for neutral images than for trypophobic images in all age groups with respect to the ‘like’ item.

These results indicate that even when using images and paradigms that differed from the approach used by Can et al.^10^, trypophobic discomfort was observed in 4–5-year-olds. In their study, children were presented with pairs of images (neutral and trypophobic images, or toxic and non-toxic images) and asked to indicate their preference; it was unclear as to what extent children experienced discomfort with each image. To our knowledge, the present study is the first developmental study to examine children’s discomfort with trypophobic images using a rating scale and direct comparison of discomfort with neutral images. In addition, ratings of the reverse item (‘like’) contrasted with ratings of the other items across all age groups, suggesting that the children understood the items they were rating and the task required. The present findings extend the results of previous studies with adult participants [e.g.,^2,4–9^] by demonstrating that children aged 4–9 years also show discomfort with trypophobic images.

In this study, we directly compared trypophobic responses between children aged 4–9 years and adults using identical items based on the Japanese version of the Trypophobia Questionnaire for adults^13^. All of the groups clearly showed more aversive responses to the trypophobic than for control images. In addition, while the average ‘disgusting’ rating for trypophobic images tended to increase with age, this was not the case for the other items. Therefore, the relationship between age and discomfort remains unclear, although our findings suggest that children, at least by the age of 4–5 years, have similar discomfort levels to adults in response to images of clustered patterns.

Notably, for some items, there were differences in the average scores by age group. For example, the scores of the 4–5-year-old children for ‘feel itchiness’ were higher than those of other age groups. One possible reason for this is that there were three negative items and one positive item; as such, participants provided three times more aversive than favorable responses, which may have influenced the overall higher ratings for ‘feel itchiness’. However, the scores for ‘like’ were also higher for 4–5-year-olds, and the adults, than those for the 8–9-year-old age group, and there were no differences in scores for any negative items other than ‘feel itchiness’ by age group. Therefore, it is difficult to explain the difference in ratings between 4 and 5-year-olds and the other age groups in terms of a response bias for aversive items.

As a limitation of this study, it is still unclear as to how discomfort in response to images of cluster patterns is acquired during development. In the future, it will be necessary to develop a method for evaluating discomfort in young children using methods other than ratings, such as non-verbal responses. For instance, Le, Cole, and Wilkins^18^ reported that adults with high trypophobic properties show an increased heart rate, heart rate variability, and blood oxyhemoglobin responses in posterior cortical areas when viewing trypophobic images, compared to neutral images. The devices they used to measure physiological responses (near infrared spectroscopy and blood flow via a pulse wave sensor) are appropriate for assessments of children, because they do not restrict movement.

In summary, the present findings suggest that trypophobic discomfort onsets at the age of 4–5 years at the latest, similar to the results of previous studies; this discomfort was not altered by direct measurement or the use of additional images. In addition, the use of a psychological scale enabled a comparison of ratings between adults and children. In the future, the development of visual preferences and discomfort in younger children and infants using various approaches should be examined to identify factors that influence trypophobic discomfort.

## Data Availability

The datasets generated and/or analyzed for the current study are available from the corresponding author on reasonable request.
